# Effectiveness of diet and physical activity interventions among Chinese-origin populations living in high income countries: a systematic review

**DOI:** 10.1186/s12889-020-08805-3

**Published:** 2020-06-29

**Authors:** Jeannette M. Beasley, Janelle M. Wagnild, Tessa M. Pollard, Timothy R. Roberts, Nasima Ahkter

**Affiliations:** 1 Department of Medicine, NYU Grossman School of Medicine, 462 First Avenue CD 673, New York, NY 10016 USA; 2 Department of Anthropology, Durham University, South Road, Durham, DH1 3LE UK; 3 NYU Health Sciences Library, NYU Grossman School of Medicine, 550 First Avenue, New York, NY 10016 USA

**Keywords:** Migrants, nutrition, Food, Exercise, Tai chi, Strength, Body mass index, Blood pressure, Lipids

## Abstract

**Background:**

This review examines the effectiveness of diet and physical activity interventions to reduce cardiometabolic risk among Chinese immigrants and their descendants living in high income countries. The objective of this review is to provide information to help build future interventions aimed at improving diet and increasing physical activity levels among Chinese immigrants.

**Methods:**

Outcomes included BMI, weight, waist circumference (WC), waist-hip ratio (WHR), cholesterol (LDL, HDL), systolic and diastolic blood pressure (SBP, DBP), hemoglobin A1c (HgbA1c), fasting blood glucose (FBG), and HOMA-IR. Six databases were systematically searched from database inception to date of search (February 2020). Meta-analyses used random effect models to estimate pooled effects of outcomes with 95% confidence intervals. The outcomes assessed were changes in mean outcomes (post-intervention versus baseline) among the intervention group versus control groups.

**Results:**

Twenty-one articles were included for synthesis, and eight of these were included in the meta-analysis. Among children/adolescents, there were no significant effects of intervention for any of the outcomes having sufficient data for meta-analysis (BMI, WHR, SBP, and DBP). Among adults, the pooled effect including three studies showed significant changes in BMI (effect size = − 1.14 kg/m^2^; (95% CI: − 2.06, − 0.21), I^2^ = 31%). There were also significant effects of intervention among adults in terms of changes in SBP and DBP, as the pooled effect across three studies was − 6.08 mmHg (95% CI − 9.42, − 2.73), I^2^ = 0% and − 3.81 mmHg (95% CI: − 6.34, − 1.28), I^2^ = 0%, respectively. Among adults there were no other significant effects among the meta-analyses conducted (weight, WC, LDL, HgbA1c, and FBG).

**Conclusions:**

This review is the first to summarize the effectiveness of diet and physical activity interventions specifically designed for Chinese immigrants living in high income countries. There were clinically meaningful changes in BMI and blood pressure among adults, but evidence was weak for other cardiometabolic outcomes (weight, WC, LDL, HgbA1c, and FBG), and among children, there was no evidence of effect for any cardiometabolic outcomes. Given our mixed findings, more work is needed to support the design of successful interventions, particularly those targeting children and their families.

**Trial registration:**

The systematic review protocol was registered in PROSPERO on December 17, 2018, the international prospective register of systematic reviews (registration number: CRD42018117842).

## Background

People of Chinese origin make up one of the fastest expanding groups in high-income countries such as the United States, Australia and Canada [1]. The cardiometabolic disease profile for this group is generally positive [1], but there are concerns about a high prevalence of type 2 diabetes identified in some studies [2, 3] and about increasing adiposity. While measures of adiposity such as BMI and waist circumference are generally low in Chinese-origin populations in high-income countries in comparison with other ethnic groups [3, 4], there is evidence that it increases with time living in a high-income country [4], that it is higher in those born to Chinese-origin parents in the United States than in migrants from China [4–6], and some evidence that it has been increasing faster amongst Chinese ethnic groups than amongst others [7]. People of Asian origin have a higher risk of cardiovascular disease at a given BMI relative to other ethnic subgroups [3], suggesting that strategies to improve diet and physical activity behaviors may be particularly important for those of Asian origin, including Chinese immigrants and their descendants [8].

There is good evidence of differences in physical activity and dietary practices between Chinese migrant groups and the rest of the population in a number of countries with the largest Chinese-origin populations. There was a higher prevalence of inactivity among Chinese Australians than non-Chinese Australians [3], Canadians of South-East Asian origin (including people with Chinese origins) were more likely to be physically inactive than the White population of Canada [9], those of Chinese origin reported lower levels of physical activity compared with the general population in the UK [10], and not only were Non-Hispanic Asians in New York City less likely to meet physical activity guidelines than non-Hispanic Whites or Blacks, but Chinese Americans were less likely to meet physical activity guidelines than other Asian subgroups [11]. Similarly, in New Zealand those of Chinese ethnicity were less likely to achieve physical activity recommendations [8].

Dietary differences are harder to characterize. Those of Chinese origin ate greater amounts of fruit and vegetables than the general population in the UK and fat intake was relatively low [12], while studies in the United States and in New Zealand found that those of Chinese ethnicity were less likely to meet recommendations for consumption of vegetables than the general population [13, 14]. Dietary patterns change with length of residence amongst migrants from China, with migrants to Canada and the United States showing negative changes such as reduced consumption of fruit and vegetables, increased portion sizes and greater consumption of convenience foods [14, 15] and a survey of Chinese immigrant mothers living in NYC reported several changes in diet after immigration including a decrease in family meals [7]. Thus interventions to promote physical activity and healthful diets could be particularly beneficial for those of Chinese-origin.

Considerations for developing interventions for Chinese migrants and/or their descendants include: 1) language (whether the intervention was offered in Cantonese, Mandarin, English, etc.); 2) health literacy; 3) traditional Chinese diet; 4) migration and acculturation; and 5) traditional Chinese medicine [16]. Successful interventions may encourage maintenance of healthful dietary practices, incorporate traditional and cultural beliefs, and provide information that would enable the participants to make healthful dietary modifications [17]. Adaptations at a surface level may involve the use of vernacular phrases, role models that represent the targeted group, identifying suitable media channels and settings for recruitment, and employing ethnically matched staff to administer the program [18]. At the deep structure level, adaptations may address the opposing cultural dimensions of collectivism and individualism [18].

In the context of some concerns about diet and physical activity in those of Chinese origin living in high-income countries, and evidence that this group may benefit from tailored interventions, this review examines the effectiveness of interventions designed to modify dietary and physical activity behaviors to reduce cardiometabolic risk in this group. The objective of this review is to provide information to build future interventions aimed at improving the diet and increasing physical activity levels among Chinese immigrants.

## Methods

The review was conducted following the PRISMA Protocol for Systematic Reviews (PRISMA) [19] and the protocol was registered in PROSPERO, International prospective register of systematic reviews (CRD42018117842).

### Information sources and search strategy

In February 2020, co-author (TR), an experienced Medical Librarian, searched PubMed Central, Ovid Medline, Ovid Embase, CABI, Food Science Technology Cinahl and the Cochrane Central Register of Controlled Trials. The Ovid Medline Search is included as supplementary material (Supplementary Table 1) to this article. The search was not limited by language or publication date. Additionally, the citations of included articles were checked and, if relevant, were included in the review.

### Eligibility criteria

This review examined diet and physical activity interventions to reduce cardiometabolic risk among Chinese immigrants living in high income countries outside of China. To this end, studies were included in the review if 1) they quantitatively described the effect of an intervention designed to modify dietary and/or physical activity behaviors on cardiometabolic risk factors (BMI, weight, waist circumference (WC), waist-hip ratio (WHR), LDL and/or HDL cholesterol, systolic and diastolic blood pressure (SBP and DBP), hemoglobin A1c (HgbA1c), fasting blood glucose (FBG), and HOMA-IR), and 2) the recipients of the intervention were of Chinese origin and living in a high-income economy, as defined by the World Bank [20]. Exclusion criteria were: studies involving institutionalized populations (as individual-level control over diet and physical activity behaviors may be restricted), and studies whose samples included residents of Hong Kong, Taiwan, and Macau (as these high-income economies are special administrative regions within China). Interventions could be at any level (individual, community, policy). The only types of studies to be excluded were observational studies in which no intervention was tested. Systematic reviews and meta-analyses on related topics were tagged for review of individual studies, but the review paper itself was not included to avoid double counting of studies. Control groups were comprised of alternative combinations of diet and physical activity interventions, attention control, cross-over designs, or before/after studies.

### Study selection and data extraction

Titles and abstracts were screened by four independent reviewers (JB, JW, TP, NA), with each citation receiving two votes. The full-texts of studies with relevant abstracts were assessed for eligibility by two screeners independently (JB, JW). Any conflicts were discussed and resolved through consensus of all four reviewers.

Data from studies eligible for inclusion were extracted using a data extraction form adapted from published sources such as the Cochrane review [21, 22]. If pre- and post-intervention means were not provided in the manuscript, the corresponding author was contacted to request the data. Quality assessment was determined using the Cochrane Review’s Risk of Bias tool [21], and guidelines provided in the Cochrane handbook for systematic reviews of interventions were used to assess risk of bias [23]. Two reviewers (JB, JW) independently extracted outcomes by reading the full articles, tables, figures and interpretations for the findings and assessed the quality of papers to ensure consistency and to minimize individual bias. Discrepancies were resolved by consensus (TP, NA, JB, JW).

### Synthesis of results

A narrative synthesis was used as it allows the compilation of data despite potential differences in research questions, design, or context in order to find a common underlying pattern. If at least two studies included the same outcome variable and pre- and post-intervention values were reported for both the intervention and control group, a meta-analysis was conducted. In cases where multiple post-intervention measurements were available, we extracted the measure that corresponded most closely to the endpoint of the intervention. We stratified analyses by age group (children/adolescents and adults).

### Statistical analysis

Where meta-analysis was possible (e.g. pre-post measures were available for intervention and control groups), the analyses involved two steps. The first step was to assess mean differences (MD) in outcomes for both the intervention and control group by comparing changes in the mean as the difference between post-intervention and baseline measures. For calculating MD, available adjusted or unadjusted means as reported in the included studies were used. The corresponding changes in standard deviation (SD) were not directly reported in most studies, and therefore was estimated using the formula suggested by the Cochrane handbook for systematic reviews of interventions [23]. A correlation of 0.6 between pre- and post-intervention values was assumed. The second step involved estimating the pooled effect for outcomes, where at least two randomized, controlled trials (RCTs) reported on the same outcome variables. The pooled effects as gain in the intervention group against the change in control group was reported as the pooled effect estimate with 95% CIs. The study weights were equal to the inverse of the variance of effect estimate of each study as suggested by DerSimonian and Laird [24, 25]. The overall effect was interpreted as statistically significant if the 95% CIs did not include the null value of 0 (no difference) in their range. Sensitivity analyses were performed to assess whether correlation of 0.5 or 0.8 affected the interpretation of the pooled effect. Heterogeneity, i.e. variation in the intervention effects observed in the included studies, was quantified using the I^2^ statistic. Results are to be interpreted with caution where there is significant heterogeneity (I^2^ > 50%). Meta-analyses were performed in R software using the ‘meta’ package.

## Results

### Study selection

**Fig. 1:**
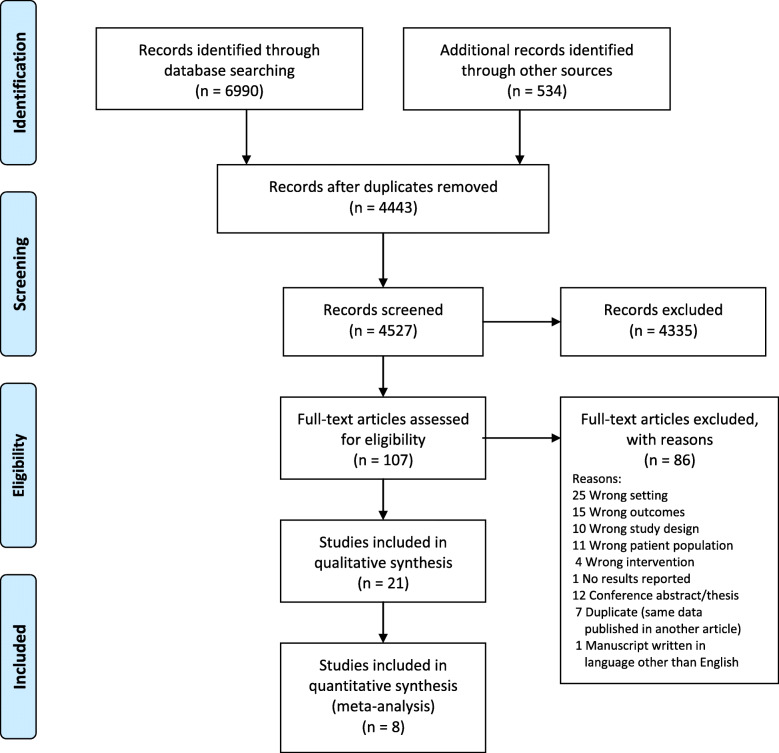
PRISMA Flow Diagram

After duplicates were removed, 4443 articles were identified (Fig. 1). The initial screening of titles and abstracts removed 4335 articles, leaving 107 full text articles to be screened by two reviewers independently (JB, JW). Of the full text articles reviewed, 86 articles were excluded for the reasons listed in Fig. 1. Twenty-one articles were included for synthesis, including one study reporting outcomes for both children and adults [26]. Of these, eight provided the pre- and post-intervention means for intervention and control groups, allowing for inclusion in the meta-analysis [26–33].

### Study characteristics

Among children/adolescents, the first study was published in 2008 [34] and the most recent study was 2019 [30] (Table 1). The range of publication dates was wider among adults (1998–2019) (Table 2). All eight studies conducted among children/adolescents were conducted in San Francisco, CA, USA [26–30, 34–36], and all but one [26] were led by the same principal investigator (Chen) (Table 1). Among adults, one study was set in Australia [37], one in Canada [33], and one in South Korea [38], while all others were conducted in the United States [31, 32, 39–47] (Table 2). The average sample size was 60 and 63 among studies conducted in children/adolescents and adults, respectively (Tables 1 and 2). The average proportion of female participants was 50 and 64.5% among studies conducted in children/adolescents and adults, respectively (Tables 1 and 2). The age range for interventions among children/adolescents was three to 18. Among children/adolescents, all interventions included both diet and physical activity components, while among adults, two interventions focused on diet exclusively while three interventions focused on physical activity exclusively (Tables 1 and 2). Among children/adolescents, intervention duration was 2 months for six studies and 6 months for two studies (Table 1). Among adults, intervention duration ranged from 5 weeks to 1 year, with most common duration of 6 months in four studies (Table 2).

**Table 1:** Study characteristics, children and adolescents

Author, year (ref)	Setting	Recruitment strategy	Data collection period	Enrollment (n)	%Female	Age range, years	Age, years (Mean, SD)	Immigration history^a^	Intervention (D, PA, D&PA)^b^	Intervention Duration
Chen 2008 [34]	Urban, San Francisco, CA, USA	Chinese community sources and after-school programs	November 2005–December 2006	57	50.9	8–10	8.8 (SD = 0.8)	NR	D&PA	6 months
Chen 2010 [27]	Urban, San Francisco, CA, USA	Chinese language programs	September 2006–December 2008	67	43.3	8–10	8.97 (SD = 0.89)	SL-ASIA: 2.38 (SD = 0.69) suggesting low acculturation	D&PA	2 months
Chen 2011 [28]	Urban, San Francisco, CA, USA	Convenience sampling from community programs	October 2007–May 2009	54	46	12–15	12.5 (SD = 3.2)	SL-ASIA: 2.13 (SD = 0.51), suggesting low acculturation	D&PA	2 months
Chen 2013 [29]	Urban, San Francisco, CA, USA	Providers in a primary care clinic recruited participants	NR	41	37	7–12	NR^a^	SL-ASIA: 1.99 (SD = 0.48), indicating low acculturation	D&PA	2 months
Chen 2015 [35]	Urban, San Francisco, CA, USA	Providers in a primary care clinic recruited participants	NR	70	~ 20	7–12	9.5 (SD = 1.6)	SL-ASIA: 2.01 (SD = 0.52), indicating low acculturation	D&PA	2 months
Chen 2016 [36]	Urban, San Francisco, CA, USA	Providers in a primary care clinic recruited participants	NR	115	30	7–12	9.5 (SD = 1.5)	SL-ASIA: 2.05 (SD = 0.56), indicating low acculturation	D&PA	2 months
Chen 2018 [30]	Urban, San Francisco, CA, USA	Two community clinics that have large Chinese American patient population recruited participants.	NR	40	42.5	13–18	14.9(SD = 1.7)	NR	D&PA	6 months
Sun 2017 [26]^c^	Urban; San Francisco, CA, USA	Four Northern California Head Start Programs in the San Francisco Bay Area:	NR	32	100	3–5	36 (SD = 4.9)	SL-ASIA: 1.92 (SD = 0.31) suggesting low acculturation.	D&PA	2 months; measurement at 0,3, and 6 months

^a^*SL-ASIA* Suinn-Lew Asian self-identity acculturation scale, ^b^*D* Diet, *PA* Physical Activity, *NR* Not reported, *SD* Standard deviation

^c^Study participants included mothers and children; children reported here

**Table 2:** Study Characteristics, Adults

Author, year (ref)	Setting	Recruitment strategy	Data collection period	Enrollment (n)	%Female	Age range, years	Age, years (Mean, SD)	Immigration history^a^	Intervention (D, PA, D&PA)^b^	Intervention Duration
Chesla 2016 [39]	Urban, San Francisco, CA, USA	Recruited through Chinese community centers, churches, grocery stores	2015	25	64	18+	57.6 (14.8) among 9 Mandarin; 54.0 (10.8) among 16 English	First-generation (*n* = 20) or second-generation (*n* = 5). SL-ASIA (Mandarin Group): 2.1 (SD = 0.5). SL-ASIA (English Group): 2.9 (SD = 0.6)	D&PA	6 months
Chiang 2009 [40]	Massachusetts, USA	Volunteers were recruited from Chinese churches, the Chinese Golden Age Center, and Chinese outpatient clinics.	NR	128	63	Age minimum was 66	73.4 (SD = 6.1)	First generation. Mean time since immigration: Culturally modified group (*n* = 58) = 21.23 years (SD = 12.89) Nonmodified group (*n* = 70) = 14.74 (SD = 9.47)	PA	2 months
Deng 2019 [41]	Urban, Greater Houston area, TX, USA	Chinese cancer survivors aged 18+ were recruited through emails, press releases, local Chinese newspapers, and announcements at local TV programs.	January 2013 to January 2014	55	78	19–91	61.7 (SD = 11.8)	First generation. Mean time since immigration: 22.2 years (SD = 11.6)	D & PA	50 weeks
Lee 2017 [38]	Urban (Korean-Chinese church and a migrant resource center); South Korea	Workers were recruited through posting and distribution of fliers at 3 Korean Chinese churches, a migrant resource center, and Korean Chinese markets. A pastor’s announcement of the study at the end of a Sunday service and word of mouth were also used to recruit participants.	January to June 2013 for the ST group and April to August 2014 for the ET group.	132	100	40–65	56.4 (SD = 5.1)	Mean duration of stay in Korea was 102.90 ± 68.08 months (about 8.5 years)	PA	6 months (3 month adoption and 3 month maintenance)
Lu 2014 [42]	Urban; Boston, MA, USA	Ads were placed in local media, and fliers were sent to neighboring primary care practices.	members of the program between January 2011–December 2011	99	58	61–83	70.6 (SD = 5.8)	NR	D&PA	6 months
Sun 2012^c^ [43]	Urban; San Francisco, CA, USA	Convenience sample of members of Chinese Community Health Partners and Chinese Community Health Research Center’s general health education program.	NR	27	52.2	NR	3 60–69 yo; 12 70–79 yo; 5 80–89 yo; 3 undisclosed	NR	D&PA	6 months
Taing 2017 [37]	Urban; Sydney, Australia	16 Mandarin-speaking general practitioners (GPs) practicing within the Central Sydney General Practice Network were recruited for the study and trained by bilingual lifestyle officers (LOs) prior to screening potential participants. The two bilingual LOs included a dietitian and an exercise physiologist that were trained in health coaching, group program delivery and standardised data collection used for evaluation. Chinese individuals were screened and referred to this study by their GP. As part of the screening and referral process, GPs administered the AUSDRISK assessment tool to determine the person’s risk of developing diabetes within five years. All individuals at high risk had blood tests to exclude undiagnosed diabetes. Those without undiagnosed diabetes who were medically cleared by their GPs were referred to the study.	NR	78	56.4	50–65	55.5(SD = 4.1)	NR	D&PA	12 months
Taylor-Piliae 2006 [44]	Urban; San Francisco, CA, USA	Subjects were recruited from the community center in cohorts, limited to 20 per group, to ensure individual attention.	NR	39	69.2	NR	65.7 (SD = 8.3)	NR	PA	3 months
Wang 2019 [45]	Urban; Midwest city, USA	Ethnically Chinese employees at an urban catering company worksite were screened for T2DM risk factors using a Chinese version of the Canadian Diabetes Risk Assessment Questionnaire (CANRISK).	NR	6	83.3	NR	NR	First generation. The majority were from mainland China and immigrated to the US within the past 5–10 years of study enrollment.	D&PA	3 months
Wang 2013 [31]	Urban; New York, NY, USA	All participants were ethnically Chinese attending a medical practice located in the neighborhood of Flushing in New York City. We screened a large database of patients attending the clinic (about 500), from which 100 patients were selected based on the exclusion/inclusion criteria detailed in methods and randomly assigned to either brown rice (n ¼ 49) or white rice (n ¼ 51) groups	NR	100	67	NR	Mean (SD) for white rice: 50 (9) and brown rice: 55 (9)	NR	D	3 months
Wang 1998 [46]	Urban; Honolulu, Hawaii, USA	Community center (“Golden Ager Association”)	NR	36	52	51–96	71.8 (SD = 9.6)	NR	D&PA	12 months
Wang 2005 [47]	Urban; Honolulu, Hawaii, USA	recruited from Chinese American social clubs, religious organizations, clinics, referrals from private physician offices, and newspaper advertisements	NR	40	51.5 (of 33 participants)	NR	68.8 (SD = 10.1)	Mean length of time in the US (*n* = 33): 16.5 (SD = 9.3)	D	10 weeks
Yeh 2016 [32]	Urban; New York, NY, USA	Chinese American Independent Practice Association (CAIPA), in collaboration with the Chinese Community Partnership for Health of New York Presbyterian-Lower Manhattan Hospital (formerly named New York Downtown Hospital).	2012–2013	60	56.7	NR	Mean (SD)Control: 60.9 (12.2) Intervention: 56.8 (9.5)	NR	D&PA	12 months
Zou 2017 [33]	Urban; Greater Toronto Area, Canada	Among the 618 Chinese Canadians who participated in blood pressure screening, 105 (17.0%) individuals were eligible to participate in this pilot trial. Among these 105 individuals, 60 (57.1%) agreed to participate and were recruited.	NR	60	51.7	NR	62.0 years (SD = 11.2)	Mean number of years living in Canada was 9.2 (SD = 6.2)	D&PA	5 weeks; pre and posttest follow-up at 8 weeks

^a^*SL-ASIA* Suinn-Lew Asian self-identity acculturation scale, ^b^*D* Diet, *PA* Physical Activity, *NR* Not reported, *SD* Standard deviation

^c^Study participants included mothers and children; mothers reported here

### Risk of bias within studies

**Fig. 2:**
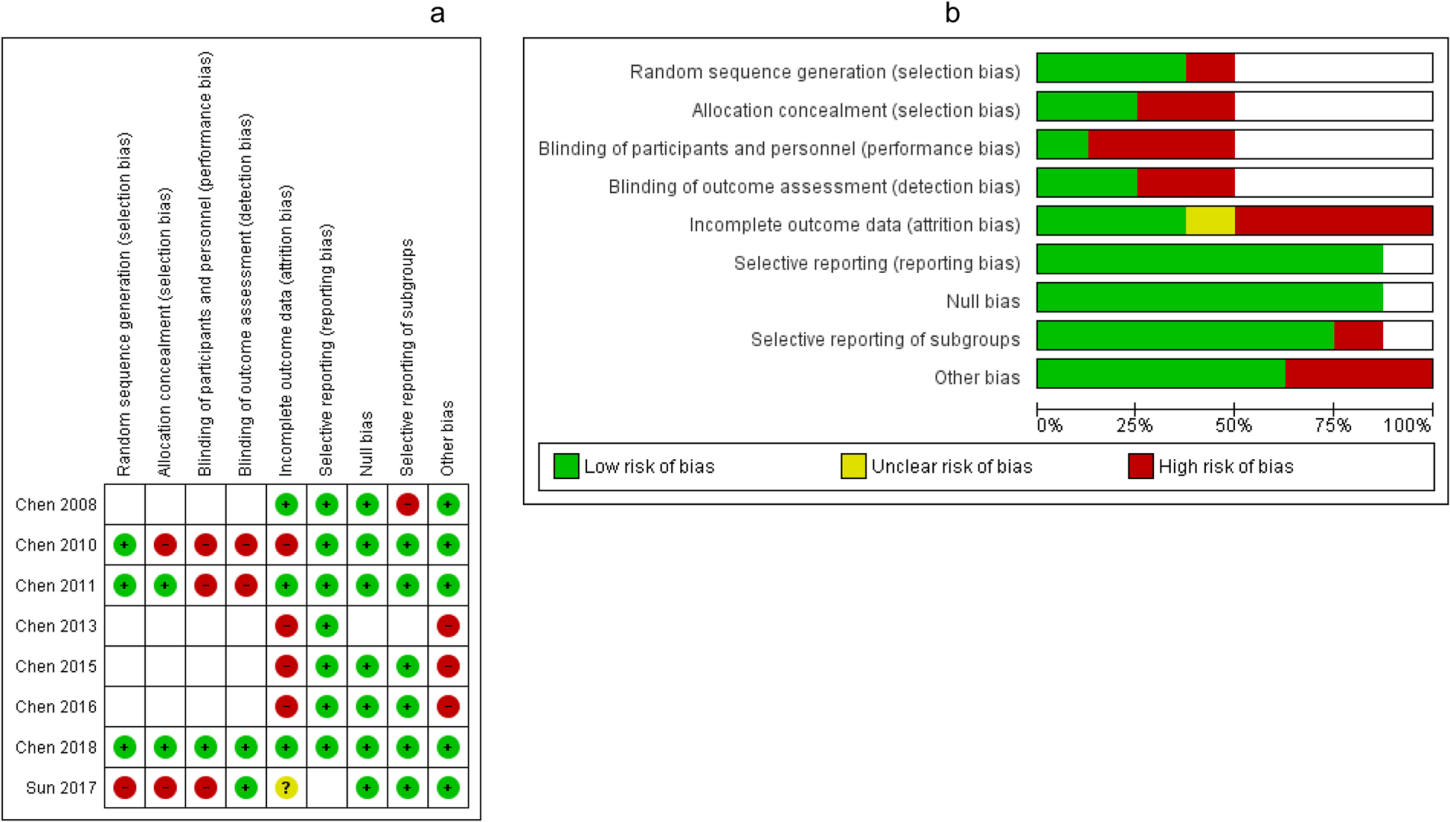
**a** and **b**. Risk of Bias Assessment, Children and Adolescents

Among studies conducted in children/adolescents (Fig. 2 and b), only Chen 2018 [30] had low risk of bias for all criteria. Four of the studies were not evaluated for random sequence generation, allocation concealment, or blinding, as they were not randomized controlled trials. Four studies had a high risk of bias for incomplete outcome data (attrition bias).

**Fig. 3:**
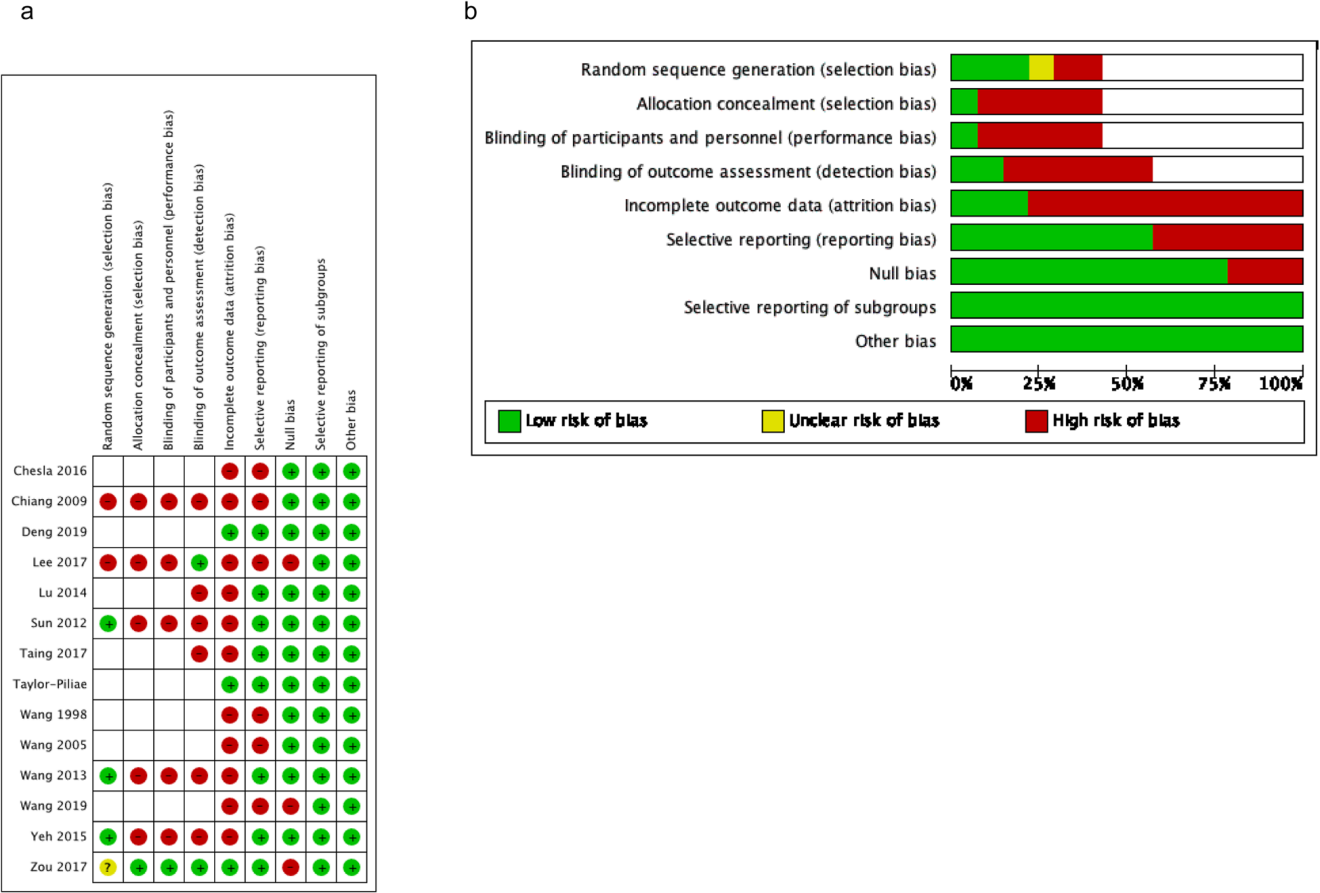
**a** and **b**. Risk of Bias Assessment, Adult

Among studies conducted in adults (Fig. 3a and b), all of the studies had at least one criterion with a high risk of bias. Six of the studies were not evaluated for random sequence generation, allocation concealment, or blinding, as they were not randomized controlled trials. Common criteria rated with a high risk of bias was blinding of outcome assessment (six studies), incomplete outcome data (ten studies), and selective reporting (five studies).

### Intervention characteristics

Among children/adolescents, four studies were randomized controlled trials, three studies were pre-post single-arm interventions, and one study included a historical control group (Table 3). The most common intervention was iStart Smart, which was adapted for Chinese American children based on the National Institute of Health’s WeCan! program (educational play-based activities teaching self-efficacy, critical thinking, and problem solving skills related to nutrition, physical activity, and coping) [29, 30, 35, 36]. Intervention components included short video clips with hands-on activities to reinforce concepts; interactive dietary software (The Wok); and 60 min exercise classes (basketball, dodge ball, badminton) weekly for 8 sessions. Study participants were provided with a pedometer, activity diary, and books related to physical activity. A one-hour parent workshop was also included to provide reinforcement and social support. Theoretical models included the Ecological Model of Childhood Obesity, Social Cognitive Theory (five studies), Transtheoretical model, and Information-Motivation-Behavior Models (Table 3).

**Table 3:** Intervention characteristics, children

Author, year (ref)	Study design^a^	Intervention group content	Comparison group content	Intervention group delivery	Comparison group delivery	Theoretical Basis	Cultural Strategies	Major Cardio metabolic Findings^b^
Chen 2008 [34]	pre-post single arm	Tailored educational materials on nutrition, physical activity, and healthy weight maintenance based on baseline assessment of their children’s weight, diet, and physical activity. Parents were instructed to follow the recommendations and share information with their children.	NA	Mothers were mailed one educational package to their homes. Researchers called parents to ensure mailed materials were understood.	NA	Ecological Model of Childhood Obesity Prevention (Davison and Birch, 2001)	Materials were modified to be compatible with Chinese and Chinese American culture. Researchers were bilingual and bicultural, and information presented to the mothers was in Chinese and English.	BMI declined significantly among children who were in the overweight category at baseline (*p* = 0.01).
Chen 2010 [27]	RCT	ABC Intervention:In sessions, children spent 15 min on physical activities and 30 min were focused on children’s knowledge regarding nutrition and physical activity and reinforced the notion of self-efficacy regarding food choices and alternatives to high-fat and high-sugar foods and television viewing. The parent intervention included a workbook, video clips and discussion of techniques.	Wait-list control group participated in data collection activities at the same time as the intervention group.	Small group weekly session activities for children, and two small group workshops for parents. Children received a food diary, books, and a weekly packet of materials.	After completing the final follow-up assessment, the control group received the ABC study intervention.	Social Cognitive Theory (Bandura)	Workshops were led by bicultural/bilingual staff. Materials were provided in both Chinese and English.	Intervention decreased body mass index and diastolic blood pressure.
Chen 2011 [28]	RCT	Web-based tailored program including activities to improve nutrition, physical activity, and coping.	Web-based general health information related to nutrition, dental care, safety, common dermatology care, and risk-taking behaviors	8 weekly online sessions for adolescents; 3 15 min lessons for parents	8 weekly online sessions for adolescents; 3 15 min lessons for parents	Transtheoretical Model–Stages of Change and social cognitive theory.	Intervention delivered in English to adolescents and in English and Chinese to parents; Interactive dietary software program (The Wok) tailored to common Chinese foods.	Intervention decreased waist-to-hip ratio and diastolic blood pressure
Chen 2013 [29]	pre-post with historical comparison group	iStart Smart (educational play-based activities teaching self-efficacy, critical thinking, and problem solving skills related to nutrition, physical activity, and coping). Short video clips with hands-on activities to reinforce concepts; Interactive dietary software (The Wok); 60 min exercise classes (basketball, dodge ball, badminton) weekly for 8 sessions; Provided pedometer, activity diary, and books related to physical activity. One 1-h parent workshop to provide reinforcement and social support.	Historical control group with weight, height, and blood pressure measured as the same interval as children in iStart Smart.	Parents and children met separately for small-group sessions. 8-weekly, 1.5 h sessions for children;a single 1-h parent workshop.	NA	Social cognitive theory (Bandura 2004)	Intervention delivered in English to children and in English and Chinese to parents.; Interactive dietary software program (The Wok) tailored to common Chinese foods	Intervention reduced BMI and BP in overweight and obese children, and improved knowledge and self-efficacy related to nutrition.
Chen 2015 [35]	pre-post single arm	iStart Smart (based on modifications to the ABC program developed previously by the first author and the national We Can! (Ways to Enhance Children’s Activity & Nutrition) program developed by the National Institute of Health)	NA	Weekly classroom activities combined with 60 min of each class in physical activity for children. Children also received a pedometer, activity diary, and books related to physical activity. They were encouraged to document their pedometer readings and challenge themselves to achieve 10,000 steps a day. Medical care was integrated into the program through individualized weight management supervised by a pediatrician at scheduled medical visits during the curriculum, and at structured follow-up intervals. The provider advised the family regarding the patient’s risk for CVD in the context of the lifestyle behaviors, laboratory values, and family history.	NA	Social cognitive theory (Bandura 2004)	Childrens’ intervention sessions were led by a bicultural, bilingual research assistant. The parent workshop was conducted in Cantonese and English and discussed both Chinese and western diets.	Average BMI percentile decreased from 94.6 (SD = 7.4) to 93.4(SD = 8.2). Similar reduction of waist/hip ratio and blood pressure were also found at 6 month follow up.
Chen 2016 [36]	pre-post single arm	Childrens’ weekly workshops included a health curriculum and physical activity. The parent workshops aimed to increase parents’ knowledge and skills regarding healthy food preparation, active lifestyle and maintaining a healthy weight tailored to the needs of each family. The program also included a field trip to a local grocery store.	NA	The children’s program included 60 min of interactive health curriculum and 60 min of physical activity each week. The parent workshop discussed both Chinese and Western diets and ways to increase physical activity in urban, underresourced communities.	NA	Social cognitive theory (Bandura 2004)	Childrens’ intervention sessions were led by a bicultural, bilingual research assistant. The parent workshop was conducted in Cantonese and English and discussed both Chinese and western diets.	Significant reduction of BMI, waist/hip ratio, and systolic blood pressure at 6-month follow-up. In addition, significant improvement of high-density lipoprotein cholesterol and decrease in triglyceride were found at 6-month follow-up.
Chen 2018 [30]	RCT	Participants (1) used a sensor to track physical activity and diet for six months, (2)reviewed eight online educational modules for three months, and then modules, (3)received tailored, biweekly text messages for three months.	Participants (1) used an OmronHJ-105 pedometer and a blank food-and-activity diary to record for three months; (2)reviewed eight online modules related to general adolescent health issues	Sequential stages; wearable sensor for 6 months, then reviewed eight online educational modules for three months, and, after completing the modules, received tailored, biweekly text messages for three months	Adolescents were asked to track and record physical activity, sedentary activity, and food intake in a diary for three months and were asked to access an online program that consisted of eight modules related to general adolescent health issues	Social cognitive theory (Bandura 2004)	Materials included concepts and beliefs with regard to promoting balance in health in Chinese and food examples that are consistent with Chinese practices and Western dietary practices.	Intervention reduced BMI, sugary beverage, TV and computer time and increased self-efficacy in nutrition and physical activity significantly more than those in the control group.
Sun 2017 [26]	RCT	Family-centered modules were developed as a tablet-based educational tool adapted from existing programs. These programs contained recommendations (5 servings fruits and vegetables, 4 cups water, 3 servings dairy, 2 h screen time, 1 h physical activity, 0 sugary drinks) for children and families to achieve a healthy lifestyle..	Weekly mailings of printed health information (e.g., food safety, choking hazards, oral health)	Intervention consisted of 8 weekly 30-min, interactive, Cantonese, educational modules delivered via tablet. Six of eight lessons were 10 to 15-min animated short videos in Cantonese, and two lessons were in a talk show format hosted by a bilingual registered dietitian.	Weekly mailings over an 8-week period	Information–Motivation– Behavior (IMB) model	Registered dietitians and health educators wrote lesson scripts in English which were then translated into Chinese by an experienced translator on the research team.	Intervention reduced maternal body mass index, waist circumference, and improved maternal eating style and self-efficacy for promoting healthy eating.

^a^*RCT* Randomized, controlled trial, ^b^*BMI* Body mass index

Among adults, three studies were randomized controlled trials, nine studies were pre-post single-arm interventions, and two studies were two-group repeated measures quasi-experimental design (Table 4). Interventions included adaptations of the Diabetes Prevention Program [32, 37, 39, 45] DASH diet [33], a cancer survival program [41], diabetes management programs [43, 46, 47], walking programs [38, 40], community-based programs [42], tai chi [44], and an intervention to incorporate more brown rice in the diet [31]. Theoretical models included Transtheoretical Model, Culture Care Theory, Chronic Care model, Theory of reasoned action, Orem’s theory of self care, Empowerment model, RE-AIM, Social Cognitive Theory, and traditional Chinese Medicine principles (Table 4).

**Table 4:** Intervention characteristics, adults

Author, year (ref)	Study design	Intervention group content	Comparison group content	Intervention group delivery	Comparison group delivery	Theoretical Basis	Cultural Strategies	Major Findings
Chesla 2016 [39]	single-group repeated-measures	Adapted Group Lifestyle Balance (GLB) curriculum: Cultural adaptation of the curriculum was conducted over 6 months by a team of nurses, a psychologist, and a social worker from a community agency that serves new Chinese immigrants.	NA	(a) a core phase, consisting of 12 weekly sessions over 3 months; (b) a transition phase, consisting of 4 sessions of decreasing frequency over 3 months	NA	NR	Cultural adaptation involved a session-by-session review of education concepts, activation strategies, and behavioral examples. Three first-generation bilingual nurse research assistants (RAs) translated the GLB participant handouts, incorporating the modifications recommended by the research team. Translations of participant handouts were checked for appropriate diabetes language and concepts by a separate community certified diabetes educator, who worked in a health agency that serves Chinese immigrants. Treatment sessions were facilitated by first generation bilingual/bicultural nurse RAs who were trained in the GLB program.	5.4% weight loss at 6 months of the study. Total and low-density lipoprotein cholesterol improved. There were no statistically significant changes in fasting plasma glucose or A1C levels.
Chiang 2009 [40]	two-group repeated measures quasi-experimental design	Walking program modified to emphasize the Chinese cultural value of authority, family members’ involvement, harmony, and balance.	Nonculturally modified walking program.	NR	NR	Transtheoretical Model and Culture Care Theory	This study intentionally added Chinese culture to only one of the groups.	The walking program had no significant effects on blood pressure or walking endurance.
Deng 2019 [41]	single-group, pre-post test design	A home-based diet and exercise intervention that was designed to improve the physical function of cancer survivors. RENEW materials were translated into Mandarin Chinese (RENEW-C) with additional PA and dietary information to ensure that the information is culturally appropriate. RENEW-C goals for each day are to (1) walk at least 30 min, (2) eat at least 3 servings of fruits, (3) eat at least 4 servings of vegetables, (4) eat no more than 20 g of saturated fat, and (5) use the “Proportion Doctor” tool.	NA	Participants engaged in a 50-week program that consisted of (1) personally tailored workbook and series of quarterly newsletters, (2) 4 consultation sessions conducted by registered dietitians who reviewed the dietary lessons and problem-solve with survivors, (3) 13 telephone counseling and 4 prompts conducted by trained LSA staff and volunteers. Phone counseling and prompts were designed to enhance social support and self-efficacy, monitor progress, identify barriers, and explore resources	NA	Social cognitive theory/ Transtheoretical model	The RENEW materials were translated into Mandarin Chinese. (RENEW-C) with additional PA and dietary information to ensure that the information was culturally appropriate. A focus group was held to evaluate the appropriateness and acceptability of RENEW-C materials. The suggested foods and corresponding caloric and fat contents in the workbook were changed to accommodate the dietary habits of Chinese Americans.	After the intervention, participants consumed higher number of servings of vegetables and engaged in PA more frequently; more participants fell within the healthy weight range. Participants showed lower limitation in doing their work or other activities due to physical health or emotional problems and encountered less experience of psychological distress and social/role incapacity.
Lee 2017 [38]	two-group, repeated measures quasi-experimental design	Over a 12 week period, 1)motivational text messages to encourage walking were sent weekly; 2) Mobile phone cartoon illustrations to help cultural adaptation were sent once every 2 weeks; 3)Participants texted the program offices every 2 weeks with their daily steps for the prior week. 4)A text message report was sent at weeks 4 and 8 with a new suggested step goal. During weeks 13–24, no intervention was provided, but women continued to text their step counts every 4 weeks.	1) Two face-to-face meetings with a nurse interventionist 2)Walking manual, a pedometer, a walking step goal, and a walking step diary. 3)Participants called the program offices every 2 weeks to report their daily steps for the prior week. 4) A registered nurse spoke to women on the phone and reviewed and adjusted their step goals at weeks 4 and 8. 5) At 12 weeks, women met with the nurse interventionist to discuss and adjust step goals. During weeks 13–24, no intervention was provided, but women continued to call their step counts every 4 weeks.	For each study arm, the principal investigator (PI) provided training sessions to the interventionists for the individual meetings based on the intervention manual. The PI directly observed the interventionist for the first 4 to 5 sessions and periodically thereafter to prevent drift. The interventionist who delivered the calls to the ST participants received training from the PI on setting step goals and how to limit the call to just providing the step goal. Individual meetings, phone calls to participants, and text messages were recorded in a spreadsheet and monitored weekly by the PI.		NR	The mobile phone cartoons were based on exit interviews with the 21 Korean Chinese women. They were asked, “What kind of information regarding everyday life would help you with adjusting to the Korean culture?” A graphic designer used the information to draw illustrations with cartoon captions of a typical daily encounter that presented an issue related to adjusting to their new culture. The illustrations included women learning about (1) the point card system utilized in a typical Korean grocery store, (2) laundering and dry-cleaning labels, (3) ordering coffee or drinks in common cafes, (4) communication skills, (5) the meaning of SPF sunscreen, and (6) nutritional values on food labels.	A significant decrease was found in 10-year risk for cardiovascular disease (CVD), blood pressure, fasting glucose, body mass index, and waist-hip ratio at weeks 12 and 24 in both groups, but there were no significant group differences.
Lu 2014 [42]	single-group repeated-measures	6-month program providing exercise, nutritional, counseling and social support to community residents with chronic diseases and mental health issues.1) 6-month membership to the YMCA was provided for a nominal fee based on income. 2) Participants met with a YMCA care manager weekly for a 1-h health education program.	NA	Half of the participants attended at least 70% of the 1-h education session with a mean attendance of 17 (63%) times out of a total of 27 sessions. Seventy-five per cent of the participants completed at least 46 exercise visits to YMCA during a 6-month period, with a mean value of 69.4 times per person, which translates into an average of 11.6 times per person per month.	NA	Wagner’s (1998) chronic Care model and multifaceted approach	1)Intervention location was convenient to elderly Chinese immigrants; 2)Bilingual and bicultural staff delivered intervention; 3) Reduced price YMCA membership to increase access for low income participants; 4)Primary care physicians referred patients to the program	Significant decrease in body weight, BMI, systolic and diastolic blood pressure.
Sun 2012 [43]	single-group repeated-measures	1) 12 biweekly 90-min support group sessions led by a multidisciplinary,bilingual team; 2) A bilingual 67-page booklet developed by CCHRC titled “Diabetes Management” was provided to participants.	NA	Program was implemented in a 6-month period. Program educators made follow-up reminder calls to encourage attendance and answer participants’ questions. Health promotion incentives were provided at no charge. A patient navigator was available 6 days per week to locate online bilingual health information for participants, provide additional guidance for utilizing glucose meters, and connect participants with potential resources that would aid in their diabetes management.A community-based participatory research approach was used to assess the effectiveness of Diabetes Self-Management.	NA	Chronic care model, Theory of reasoned action, and Social Cognitive Theory	All instructional materials were written at a Chinese layman fourth-grade level. To ensure information was culturally appropriate, program educators incorporated Chinese commonly practiced activities and food items into the educational curriculum and in-person sessions. The class curriculum and handouts were focus group tested with the target population. Classes were held in a medical office building in San Francisco Chinatown, all activities were conducted in Cantonese, and participants were given a bilingual book on diabetes management.	Statistically significant increases in glycemic control and diabetes knowledge. At 6 months after enrollment, 42.1% (*n* = 8) of the participants had a clinical significant glycemic control improvement by achieving ≥1.0% decrease in A1C; 31.6% (*n* = 6) had slight improvements in A1C (< 1.0% decrease); and 26.3% (n = 5) had no improvement or increase in A1C (≤ 0.0% decrease) from baseline. Statistically insignificant differences were shown in diabetes management practices. Secondary outcomes assessed participants’ perceived diabetes management and emotional and social support
Taing 2017 [37]	single-group repeated-measures	Promoted: 1) Increasing amount of moderate to vigorous intensity aerobic (150 min/week) and progressive resistance training (60 min/week) to 210 min/week; 2) Reducing percent total energy from fat and saturated fat intake to less than 30 and 10%, respectively; 3) Consuming at least 15 g/1000 kcal of dietary fiber intake; 4) Reducing body weight by 5% after 12 months.	NA	Assisted telephone interview survey was completed at baseline and 12 months; 2)1.5-h individual initial consultation with interventionist. 2) Three 2-h lifestyle group sessions;3) Three follow-up health coaching phone calls, lasting 20–30 min each, at 3, 6, and 9-months 4) Face-to-face individualreview at 12 months	NA	NR	Consultations with an Advisory Group resulted in 1) Conducting the program entirely in Mandarin; 2)Translating all resources and materials to Mandarin; 3), Having two bilingual interventionists.	Waist circumference, total cholesterol and fat intake significantly improved at 12-months.
Taylor-Piliae 2006 [44]	single-group repeated-measures	Yang Style 24-posture short-form Tai Chi was taught by an instructor with experience teaching olderadults. The Yang Style 24-posture short-form is easier to learnand remember than the classical Yang style 108-posture long form, though still contains the essential Tai Chi principles.	NA	1) 60-min Tai Chi exercise class 3 times per week for 12 weeks, located at the community center; 2) Instruction to practice at home at least twoother days; 3) CD-Romof the instructor performing Tai Chi given at 12-weeks. Subjects were monitored for safety with corrections given as needed.	NA	NR	Culturally relevant and appropriate forms of physical activity and exercise may contribute to better adherence. Tai Chi is a traditional form of exercise among Chinese populations. Intervention was offered at community center in both English and Cantonese	Clinically and statistically significant reductions in blood pressure at rest (131/77), and in response to the step-test (164/82) were found over 12 weeks of TC (*p* < 0.01). No significant change in heart rate was observed.
Wang 2019 [45]	single-group, pre-post test design	A modified and tailored 12-week, DPP lifestyle modification course was developed based on identified topics from Chinese employees	NA	The course was adjusted to be delivered weekly on an individual basis to accommodate different work schedules. The 12-week course was delivered in Chinese by the project leader; educational materials in Chinese were handed out at each session to facilitate learning. The course was convened generally during the first shift’s lunch break or before the beginning of the second shift.	NA	NR	Educational materials were translated into Chinese and adjusted to use common words, avoid medical vocabulary, break down long sentences to short phrases, and include pictures to facilitate learning.	Participants showed an average reduction of nonfasting blood glucose of 30 mg/ dL (1.7 mmol/L), and a reduction of HbA1c by 0.32 points (3 mmol/mol).
Wang 2013 [31]	RCT	For each study arm (brown and white rice), all subjects were provided free rice. Subjects were encouraged to prepare rice items in their daily meals with the food items provided for the duration of the study and they were also advised not to change their usual patterns of cooking and eating.		For each study arm, the supplies provided were enough to meet the calculated total energy requirements for a 4-week period. No rice was provided for the family or other household members.		NR	NR	Significant decreases in weight and systolic and diastolic blood pressure among brown rice (intervention) group only. Insulin and HOMA, serum AGEs and 8-isoprostane decreased, while SIRT1 mRNA increased in the brown rice group as compared to the white rice group
Wang 1998 [46]	single-group, pre-post test design	Consultation with a diabetes nurse educator for an individualized meal plan, exercise plan, preventive plan for hyperglycemia and hypoglycemia, and foot care.	NA	Counseling by diabetes nurse educator; bi-weekly checks of blood pressure and/or blood glucose for one year	NA	Orem’s theory of self-care	Conducted in Chinese; individualized meal plan per dietary preferences	Eighty percent of participants had decreased their diastolic blood pressure from above 95 mmHg to below 90 mmHg and systolic blood pressure from above 155 mmHg to below 140 mmHg. Range of participants’ blood glucose levels also decreased from 126 mg/dL – 277 g/dL to 85 mg/dL – 226 mg/dL after participating in the program
Wang 2005 [47]	single-group repeated-measures	Intervention topics included 1) Nutrition 2) Exercise 3) Medication compliance; 4)Stress management; and 5) Foot and skin care activities.	NA	During the 10 weeks of the program, four sessions were offered on different days of the week to accommodate participants` schedules. The investigator and a registered nurse delivered the group sessions for up to 10 people.	NA	Empowerment model	Classes were conducted in Cantonese, Mandarin, or Taiwanese. Because the Chinese translation for diabetes is sugar urine disease, many participants took the term literally and thought that they had to avoid only sweet tasting foods. Many participants reported that theirphysicians instructed them to consume less rice; subsequently, some participants avoided rice but consumed other carbohydrates (e.g., noodles or buns). Hence, the dietary education component of the program emphasized the concept of carbohydrates.	43.6% of the participants lost more than 5 poundsand most had a reduction in blood pressure at 3 months after completion of the program. Mean HbA1c decreasedfrom 7.11 to 6.12 post-intervention.
Yeh 2016 [32]	RCT	The Diabetes Prevention Program curriculum was adapted based on feedback from three focus groups of Chinese participants with pre-diabetes and one advisory group meeting.	Diabetes prevention information provided through mailings	12 bi-weekly core sessions and six monthly follow-up sessions conducted by trained lifestyle coaches at a community site that could accommodate an exercise program.	Quarterly mailings	RE-AIM	Sessions were conducted in Mandarin or Cantonese. Sessions were adapted to include more information about Asian diabetes risk disparity, following each intervention with a physical activity session (e.g. walking group or tai chi), inviting family members to attend sessions, providing measuring cups (especially rice bowls for portion control), as well as culturally and linguistically tailoring.	There was a significantly greater percent weight loss in the intervention group (3.5 vs. 0.1%; *P* = 0.0001) at 6 months, which was largely maintained at 12 months (3.3 vs. 0.3%; *P* = 0.0003).
Zou 2017 [33]	RCT	Intervention components were usual care plus (1) the DASH diet pattern (2) sodium reduction; (3) Traditional Chinese Medicine food therapy	Usual care consisted of: (1)hypertension health education booklet; (2) encouragement to see their primary health care provider regarding their blood pressure; (3) information on how to access local healthcare services	(1) Intervention Manual and a refrigerator poster to summarize the dietary recommendations; (2) two 2-h classroom sessions; (3) 20-min booster telephone call 5 weeks post-randomization	Information provided at baseline	Traditional Chinese Medicine (TCM) principles of TCM food therapy: (1) light eating; (2) balance between the hot and cold nature of food; (3) harmony of the five flavors of food (sour, sweet, bitter, pungent and salty); and (4) consistency of diet withvarious health conditions.	Intervention sessions delivered in Mandarin; incorporated Traditional Chinese Medicine into intervention components	At 8 weeks post-randomization, those in the intervention group had greater reductions in systolic blood pressure [3.8 mmHg, t (55) = − 1.58, *p* = 0.12] compared to those of the control group.

### Intervention effectiveness

**Fig. 4:**
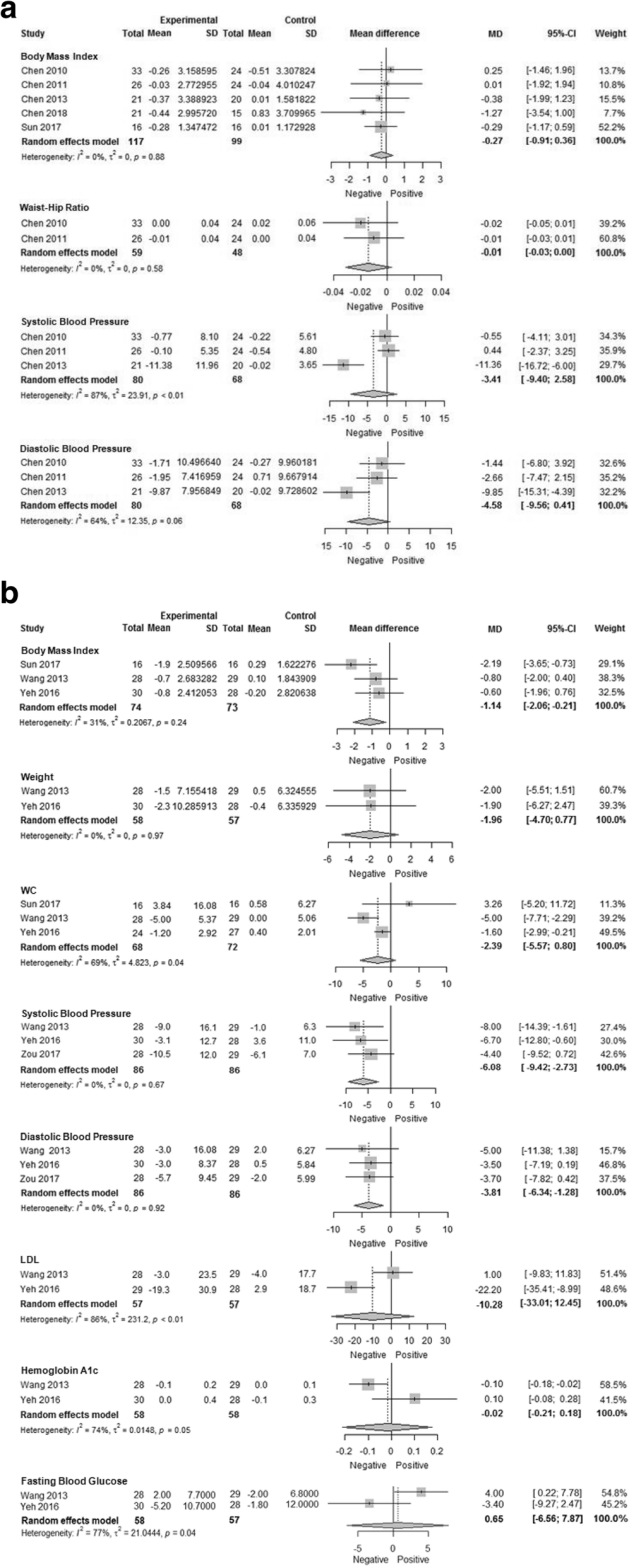
**a** Meta-analysis of mean change in cardiometabolic outcomes from baseline to post-intervention for Chinese migrant children/adolescents. **b** Meta-analysis of mean change in cardiometabolic outcomes from baseline to post-intervention for Chinese migrant adults

Among children/adolescents, sufficient data were available for meta-analysis for BMI, WHR, SBP, and DBP. The pooled effect including five studies did not show significant changes in BMI (effect size = − 0.27 kg/m^2^; (95%CI -0.91, 0.36) (Fig. 4a). For WHR, there were also no significant changes over time between groups, (two pooled studies with an effect size − 0.01 (95%CI -0.03, 0.00). There was also no significant effect of intervention in terms of changes in SBP or DBP as the pooled effect across three studies was − 3.41 mmHg (95%CI -9.40, 2.58) and − 4.58 mmHg (95%CI -9.56, 0.41), respectively. Results did not substantively change in sensitivity analyses using 0.5 and 0.8 as the correlation between baseline and follow-up measures (data not shown). For the other outcomes of interest (WC, LDL, HDL, and FBG) (Table 5), just one study reported findings, and statistically significant differences were only reported for HDL.

**Table 5:** Cardiometabolic outcomes- children

		Intervention group						Control group					
		Baseline			Post-intervention			Baseline			Post-intervention		
	Author, year	Mean	SD	n	Mean	SD	n	Mean	SD	n	Mean	SD	n
BMI (kg/m^2^)	Chen 2010	19.74	3.58	35	19.48	3.48	33	18.65	2.63	32	18.14	2.60	24
	Chen 2011	20.79	3.12	26	20.76	3.08	26	20.25	3.21	24	20.21	3.13	24
	Chen 2013	25.53	3.65	21	25.16	3.91	21	23.17	1.22	20	23.18	1.28	20
	Chen 2015	24.03	3.47	70	23.67	3.52	70						
	Chen 2016	23.7	3.6	115	23.4	3.5	115						
	Chen 2018	27.37	3.26	23	26.93	3.43	21	28.35	4.36	17	29.18	3.88	15
	Sun 2017	16.86	1.57	16	16.58	1.43	16	16.24	1.28	16	16.25	1.34	16
WC (cm)	Chen 2013	82.63	11.25	21	81.33	10.77	21						
WHR	Chen 2010	0.88	0.04	35	0.88	0.04	33	0.89	0.06	32	0.91	0.06	24
	Chen 2011	0.91	0.04	26	0.90	0.04	26	0.89	0.04	24	0.89	0.04	24
	Chen 2015	0.92	0.06	70	0.91	0.06	70						
	Chen 2016	0.95	0.09	115	0.94	0.09	115						
LDL (mg/dL)	Chen 2016	101.92	34.23	115	100.69	36.29	115						
HDL (mg/dL)	Chen 2016	47.83	10.39	115	50.94	10.24	115						
SBP (mmHg)	Chen 2010	105.74	9.01	35	104.97	9.10	33	99.87	5.81	32	99.65	6.63	24
	Chen 2011	102.02	5.9	26	101.92	6.05	26	101.13	4.55	24	100.59	5.86	24
	Chen 2013	106.9	5.75	21	95.52	14.49	21	101.33	4.56	20	99.64	2.80	20
	Chen 2015	104.5	8.8	70	98.3	11.8	70						
	Chen 2016	104	8.8	115	99.8	10.9	115						
DBP (mmHg)	Chen 2010	63.23	12.91	35	61.52	9.62	33	57.70	11.31	32	57.43	10.95	24
	Chen 2011	63.26	8.19	26	61.31	8.39	26	60.43	9.98	24	61.14	11.44	24
	Chen 2013	62.73	7.11	21	52.86	9.83	21	59.92	11.2	20	59.27	10.51	20
	Chen 2015	61.9	8.7	70	57.0	12.1	70						
	Chen 2016	62.7	8.3	115	59.1	11.1	115						
FBG (mg/dL)	Chen 2016	85.89	5.24	115	85.52	6.21	115						

For the three single group design studies, Chen 2008 only reported changes in BMI stratified by overweight status [34], while the other two reported minor improvements in BMI and blood pressure (Table 5) [35, 36].

Among adults, sufficient data were available for meta-analysis for BMI, weight, WC, SBP, DBP, LDL, HgBA1c, and FBG. The pooled effect including three studies showed significant changes in BMI (effect size = − 1.14 kg/m^2^; 95%CI − 2.06, − 0.21) (Fig. 4b). In contrast, among the two studies reporting weight, the effect was null (effect size = − 1.96 kg; 95%CI -4.70, 0.77). For waist circumference, there were also no significant changes over time between groups (three pooled studies with an effect size − 2.39 (95%CI -5.57, 0.80)). There were significant effects of intervention in terms of changes in SBP and DBP, as the pooled effect across three studies was − 6.08 mmHg (95%CI − 9.42, − 2.73) and − 3.81 mmHg (95%CI − 6.34, − 1.28), respectively. Finally, there was no significant effect of intervention on LDL (effect size = − 10.28 mg/dL; 95%CI -33.01, 12.45), HgBA1c (effect size = − 0.02%; 95%CI -0.21, 0.18), or FBG (effect size = 0.65 mg/dL; 95%CI -6.56, 7.87). Results did not substantively change in sensitivity analyses using 0.5 and 0.8 as the correlation between baseline and follow-up measures (data not shown).

For the eleven studies that were not randomized controlled trials (Table 6), minor improvements were documented in BMI, weight, LDL, SBP, DBP, FBG, and HgbA1c. However, without a rigorous comparison group, the effects cannot be attributed to the interventions delivered with certainty. Data from one of the studies was not included in Table 6 due to incompatibility of the scales used to measure outcomes [45].

**Table 6:** Cardiometabolic outcomes- Adults

		Intervention group						Control group					
		Baseline			Post-intervention			Baseline			Post-intervention		
	Author, year	Mean	SD	n	Mean	SD	n	Mean	SD	n	Mean	SD	n
BMI (kg/m^2^)	Chesla 2016	29.4	3.6	25	27.5	4.5	25						
	Deng 2019	23.2	3.6	50	23.86	4.5	50						
	Lu 2014	25.1	3.4	98	24.7	3.3	88						
	Sun 2017	24.67	2.89	16	22.77	2.71	16	25.3	2.57	16	25.59	2.56	16
	Wang 2013	26.5	3	28	25.8	3	28	24.9	2	29	25	3	29
	Yeh 2016	26.3	2.4	30	25.5	2.9	30	25.8	2.3	30	25.6	4.3	28
Weight (kg)	Chesla 2016	78.1	12.8	25	73.0	13.6	25						
	Lu 2014	64.1	9.5	98	63.2	9.4	88						
	Taing 2017	66.9	9.4	78	-0.5	0.4	78						
	Wang 2005	63.3	12.1	33	55.8	22.2	33						
	Wang 2013	64.9	8	28	63.4	8	28	63.3	10	29	63.8	10	29
	Yeh 2016	69.9	11.5	30	67.6	11.5	30	66.4	9.8	28	66	10.2	28
WC (cm)	Sun 2017	86.33	8.69	16	90.17	19.71	16	85.04	6.45	16	85.62	7.44	16
	Wang 2013	87	6	28	82	6	28	84	8	29	84	8	29
	Yeh 2016	36.1	3.4	24	34.9	3.1	30	35.3	3.3	27	35.7	3	28
WHR													
LDL (mg/dL)	Chesla 2016	114.6	36.8	25	98.8	28.7	25						
	Taing 2017	3.2	0.9	74	-0.36	0.1	74						
	Wang 2013	101	28	28	98	24	28	104	20	29	108	29	29
	Yeh 2016	107.2	38.1	30	87.9	27.7	29	108.1	30.6	30	91.2	27.8	28
HDL (mg/dL)	Wang 2013	51	14	28	52	12	28	55	16	29	54	14	29
SBP (mmHg)	Lu 2014	130.2	12.3	98	124.6	9.8	88						
	Taylor-Piliae 2006	150	20	38	131.1	15.1	38						
	Wang 1998	155.1	15.9	75	142.8	15.3	75						
	Wang 2005	131.5	13.6	33	118.9	42.1	33						
	Wang 2013	123	10	28	114	13	28	118	12	29	118	18	29
	Yeh 2016	127.1	13.6	30	124	14.7	30	126.6	18.3	30	125.2	15.8	28
	Zou 2017	145.6	11.1	28	135.1	14.7	28	146.4	8.6	29	139.7	11.6	29.000
DBP (mmHg)	Chesla 2016	82.2	12.2	25	78.4	7.1	25						
	Lu 2014	79.2	8	98	76.1	7.2	88						
	Taylor-Piliae 2006	85.8	9.3	38	76.9	8.4	38						
	Wang 1998	93.1	4.2	75	83.1	5.8	75						
	Wang 2005	69.4	10.9	33	63.4	23.4	33						
	Wang 2013	75	6	28	72	6	28	75	8	29	76	8	29
	Yeh 2016	78.6	9.5	30	75.6	9.2	30	78.1	9.7	30	74.8	8.3	28
	Zou 2017	90.5	7.5	28	84.8	11.8	28	87.6	9.8	29	84.5	9	29
HgBA1c	Chesla 2016	5.91	0.27	25	5.89	0.2	25						
	Sun 2012	7.87	0.97	19	7.11	0.62	19						
	Wang 2013	5.9	0.2	28	5.8	0.2	29	5.8	0.2	29	5.8	0.2	29
	Wang 1998	7.11	1.1	33	6.12	2.4	33						
	Yeh 2016	6.2	0.4	30	6.2	0.4	30	6	0.3	30	6.2	0.5	28
FBG (mg/dL)	Chesla 2016	96.4	6.7	25	93.1	5.6	25.000						
	Wang 2013	91	8	28	93	9	28	91	8	29	89	7	29
	Yeh 2016	109.7	8.8	30	104.5	13.3	30	103.3	11.7	30	101.5	14.5	28
HOMA-IR	Wang 2013	1.5	1.2	28	1.3	1.2	28	1.1	1	29	1.1	0.8	29

*Results from Taing 2019 omitted, as post-intervention means and standard deviations weren’t provided by the authors. Yeh 2016 results were obtained from the lead author

## Discussion

As of February 2020, there were 21 published studies describing behavioral diet and physical interventions in Chinese migrants living in high-income countries. The majority were conducted in adults (*n* = 13), and just three of the adult interventions were conducted outside the United States (Australia, Canada, South Korea). Eight were conducted in children/adolescents; of these, seven were conducted by the same research group in San Francisco.

There were clinically meaningful changes in BMI [48] and blood pressure [49] among adults, but evidence was weak for other cardiometabolic outcomes (weight, WC, LDL, HgbA1c, and fasting glucose), and among children, there was no evidence of effect for any cardiometabolic outcomes. The intervention having the largest change in BMI among adults (− 2.19) had a much smaller effect on the offspring (− 0.29) [26]. Several explanations may help explain the differences in effects observed between adults and children in this study and others. First, post-intervention measures were collected 3 months later in children, while mothers’ BMI was collected immediately following the intervention. Second, BMI z-scores, which better account for growth stage compared to BMI among children, were not reported by the authors. Furthermore, most of the adult intervention periods were longer-term (6–12 months) whereas most of the studies conducted among children were 2 months in duration.

This report fills a gap in our understanding of the evidence base for behavioral diet and physical activity interventions conducted in Chinese migrants and their descendants living in high-income countries. Other reviews have examined diet and physical activity behaviors among African [50] and South Asian [51] migrants to high-income countries. For example, a review of the effects of diet and physical activity interventions on weight, BMI, and waist circumference among South Asian migrants including 29 studies also observed no significant differences among children but a significant improvement in weight only among adults (mean difference − 1.8 kg, 95% CI − 2.5 to − 1.2 kg) [51].

Limitations must be acknowledged in interpreting these findings. Despite searching seven databases and reference lists for all identified articles, it is possible that relevant studies were missed, if for example, the title or abstract didn’t describe analyses specific to Chinese migrants. Although the characteristics of each intervention as are described in this review in order to help identify which intervention components might be effective, given the small sample size and heterogeneity of the studies, the review cannot definitively summarize successful strategies for behavioral diet and physical activity interventions targeted at Chinese-origin groups [52–55].

Most studies conducted a complete case analysis rather than accounting for loss to follow-up incorporating missing data methods such as multiple imputation. Complete case analyses would overestimate any effect of the intervention if, for example, participants who dropped out lost less weight compared to those who completed the study. We did not make any adjustment for how studies accounted for attrition in our analysis, but attrition bias was accounted for in the quality assessment. In summary, a major limitation of our analyses was having a relatively small number of controlled trials that were suitable for meta-analyses. We only included controlled trials, as opposed to single arm pre-post studies, in the meta-analyses to minimize the likelihood that observed changes in cardiometabolic outcomes were due to factors other than the intervention, particularly in growing children.

Suggestions for improvement include increased attention to (1) how interventions are culturally adapted; (2) the types of behavior change techniques and theories that are used to underpin interventions; (3) loss to follow-up by study arm; (4) variability within the Chinese-origin population, particularly with respect to generational differences that may be important for the design of interventions; and (5) contextual factors, such as whether the setting is rural or urban. These recommendations would enable reviewers to assess how behavior change techniques and theories moderate effectiveness, to assess the equity impacts of interventions, and to examine explanations for heterogeneity between interventions.

## Conclusions

Given our mixed findings, more work is needed to support the design of successful interventions, particularly those targeting children and their families. The development of effective interventions may well require a great deal of qualitative and quantitative research on knowledge, attitudes, behaviors, and perceptions. More research is needed into the differential effects of lifestyle interventions for Chinese immigrants compared with other ethnicities.

## Supplementary information

**Additional file 1: Supplemental Table 1** Ovid Medline Database Search Strategy.

## Data Availability

The datasets used and/or analysed during the current study are available in the published literature.

## Abbreviations

BMI: Body mass index
CA: California
CABI: Commonwealth Agricultural Bureaux International
CI: Confidence Interval
D: Diet
DASH: Dietary Approaches to Stop Hypertension
DBP: Diastolic blood pressure
FBG: Fasting blood glucose
HDL: High density lipoprotein
HgbA1c: Hemoglobin A1c
HOMA-IR: Homeostatic Model Assessment of Insulin Resistance
kg: Kilogram
LDL: Low density lipoprotein
m: Meter
mg/dL: Milligram per deciliter
mmHg: Millimeters of mercury
MD: Mean difference
NR: Not reported
NYC: New York City
PA: Physical activity
PRISMA: Preferred Reporting Items for Systematic Reviews and Meta-Analyses
PROSPERO: PROSPective Register Of systematic reviews
RCT: Randomized, controlled trial
RE-AIM: Reach, Effectiveness, Adoption, Implementation, Maintenance
SBP: Systolic blood pressure
SD: Standard deviation
SL-ASIA: Suinn-Lew Asian self-identity acculturation scale
UK: United Kingdom
USA: United States of America
WC: Waist circumference
WHR: Waist hip ratio
