# Supplementary material for: Effectiveness of diet and physical activity interventions among Chinese-origin populations living in high income countries: a systematic review
Source: BMC Public Health. 2020 Jun 29;20:1019. doi: 10.1186/s12889-020-08805-3 (PMC7322842; doi:10.1186/s12889-020-08805-3)
Supplement: Supplementary file 1 — Additional file 1: Supplemental Table 1 Ovid Medline Database Search Strategy. [file 12889_2020_8805_MOESM1_ESM.docx]

**Supplemental Table 1**

Ovid Medline Database Search Strategy

Searched February 22, 2020

| 1. exp Healthy Lifestyle/ or (health* adj3 (lifestyle* or diet* or behavio* or promotion* or educat*)).tw. |  |
| --- | --- |
| 2. diet/ or diet, carbohydrate loading/ or diet, diabetic/ or exp diet, carbohydrate-restricted/ or diet fads/ or diet, fat-restricted/ or diet, gluten-free/ or diet, high-fat/ or exp diet, high-protein/ or diet, mediterranean/ or diet, paleolithic/ or diet, protein-restricted/ or diet, reducing/ or diet, sodium-restricted/ or exp diet, vegetarian/ or diet, western/ or dietary approaches to stop hypertension/ or healthy diet/ or portion size/ or serving size/ |  |
| 3. ((Portion or Serving) adj1 Size*).tw. |  |
| 4. (Diet* adj1 (Fad* or Carbohydrate* or Diabet* or Fat* or Gluten or Protein or Mediterranean or Paleolithic or Reducing or Sodium or Vegetarian or Macrobiotic or Vegan or Western or Calori*)).tw. |  |
| 5. exp health promotion/ |  |
| 6. exp Overweight/dh, pc or exp Obesity/dh, pc |  |
| 7. exp Exercise/ or exp Exercise Therapy/ or (Exercise* or Running or Jog* or Swim* or Walk* or Aerobic*).tw. or (Physical adj1 (exert* or Activit*)).tw. |  |
| 8. exp Sports/ or sport*.tw. |  |
| 9. exp Exercise Movement Techniques/ or (Pilates or Qigong or Yoga or Tai-ji or Tai Chi or Tai Ji or Taiji or Taijiquan or T'ai Chi or Qi Gong or Ch'i Kung).tw. |  |
| 10. exp Dietary Carbohydrates/ or exp Dietary Fats/ or exp Dietary Fiber/ or exp Dietary Proteins/ or exp Dietary Supplements/ or exp Nutritional Requirements/ or Nutritional Status/ or Nutrition Therapy/ or (nutrition* adj1 (educat* or promot* or therap*)).tw. |  |
| 11. exp Patient education as topic/ |  |
| 12. exp "Nutritional and Metabolic Diseases"/dh, pc or exp Diabetes Mellitus/dh, pc |  |
| 13. exp Cardiovascular Diseases/dh, pc |  |
| 14. 1 or 2 or 3 or 4 or 5 or 6 or 7 or 8 or 9 or 10 or 11 or 12 or 13 |  |
| 15. (Asian Continental Ancestry Group/ or Asian Americans/) and chinese.tw. |  |
| 16. China/eh |  |
| 17. ((cantonese or Mandarin or Hmong) adj1 speak*).tw. |  |
| 18. ((China or Chinese or Han or Mandarin or Hmong* or Canton*) adj1 (America* or Canad* or Australia*)).tw. |  |
| 19. (((((((Andorra or Germany or Oman or Antigua or Barbuda or Gibraltar or Palau or Argentina or Greece or Panama or Aruba or Greenland or Poland or Australia or Guam or Portugal or Austria or Hong Kong or Puerto Rico or Bahamas or Hungary or Qatar or Bahrain or Iceland or San Marino or Barbados or Ireland or Saudi Arabia or Belgium or Isle of Man or Seychelles or Bermuda or Israel or Singapore or British Virgin Islands or Italy or Saint Maarten or Brunei Darussalam or Japan or Slovak Republic or Canada or Korea or Slovenia or Cayman Islands or Kuwait or Spain or Channel Islands or Latvia or Saint Kitts) and Nevis) or Chile or Liechtenstein or Saint Martin or Croatia or Lithuania or Sweden or Curacao or Luxembourg or Switzerland or Cyprus or Macao or Taiwan or Czech Republic or Malta or Trinidad) and Tobago) or Denmark or Monaco or Turks) and Caicos) or Estonia or Netherlands or United Arab Emirates or Faroe Islands or New Caledonia or United Kingdom or Finland or New Zealand or United States or France or Northern Mariana Islands or Uruguay or French Polynesia or Norway or Virgin Islands).mp. [mp=title, abstract, original title, name of substance word, subject heading word, floating sub-heading word, keyword heading word, protocol supplementary concept word, rare disease supplementary concept word, unique identifier, synonyms] |  |
| 20. (United States or America* or USA or Alabama or Alaska or Appalachian Region or Arizona or Arkansas or California or Colorado or Connecticut or Delaware or District of Columbia or Florida or Georgia or Great Lakes Region or Hawaii or Idaho or Illinois or Indiana or Iowa or Kansas or Kentucky or Louisiana or Maine or Maryland or Massachusetts or Michigan or Mid-Atlantic Region or Midwestern United States or Minnesota or Mississippi or Missouri or Montana or Nebraska or Nevada or New England or New Hampshire or New Jersey or New Mexico or New York or North Carolina or North Dakota or Northwestern United States or Ohio or Oklahoma or Oregon or Pacific States or Pennsylvania or Rhode Island or South Dakota or Southwestern United States or Tennessee or Texas or Utah or Vermont or Virginia or Washington or West Virginia or Wyoming).mp. |  |
| 21. "Emigrants and Immigrants"/ or Acculturation/ or Ethnicity/ or Ethnic groups/ |  |
| 22. ((China or Chinese or Han or Mandarin or Hmong* or Canton*) adj1 (ethinic* or ancest* or immigrant* or emigrant* or migrant* or acculturat* or subgroup* or relocat* or population* or communit* or international* or America* or Canad* or Australia*)).tw. |  |
| 23. 15 or 16 or 17 or 22 |  |
| 24. (ethinic* or ancest* or immigr* or emigr* or migrant* or acculturat* or subgroup* or relocat* or population* or communit* or international*).tw. |  |
| 25. 18 or 19 or 20 or 21 or 24 |  |
| 26. 23 and 25 |  |
| 27. 14 and 26 |  |
